# Apolipoprotein E polymorphism is associated with lower extremity deep venous thrombosis: color-flow Doppler ultrasound evaluation

**DOI:** 10.1186/1476-511X-13-21

**Published:** 2014-01-24

**Authors:** ShuangLi Zhu, ZhiGang Wang, XiaoPing Wu, Yan Shu, DunXiang Lu

**Affiliations:** 1 Institute of Ultrasound Imaging, The Second Affiliated Hospital, Chongqing Medical University, 76 Riverside Road, Yuzhong District, Chongqing 400010, P. R. China; 2 Institute of Ultrasound Imaging, Mentougou Hospital, Beijing 102300, P. R. China; 3 Institute of Ultrasound Imaging, The First Affiliated Hospital of Inner Mongolia Medical University, Huhhot 010059, China

**Keywords:** Apolipoprotein E, Gene polymorphism, Lower extremity deep venous thrombosis, Case–control study

## Abstract

**Introduction:**

Apolipoprotein E (apoE) is a member of apolipoprotein family, and its gene polymorphisms seem to have some impact among patients with cardiovascular disease. However, its role in the lower extremity deep venous thrombosis (LEDVT) has not been well studied. The objective of this study was to investigate the potential association between *APOE* gene polymorphisms and LEDVT.

**Materials and methods:**

A hospital-based case–control study was conducted in 300 patients with LEDVT by color-flow Doppler ultrasound and 300 age- and gender-matched healthy controls. Polymerase chain reaction restriction fragment length polymorphism (PCR-RFLP) assay was applied to assess the *APOE* gene polymorphisms.

**Results:**

Patients with LEDVT had a significantly higher frequency of *APOE* E3/E4 genotype [odds ratio (OR) =1.48, 95% confidence interval (CI) = 1.05, 2.10; *P* = 0.03] than healthy controls. When stratifying by family history of LEDVT, it was found that patients with positive family history of LEDVT had a significantly higher frequency of *APOE* E3/E4 genotype (OR =1.68, 95% CI = 1.04, 0.95; *P* = 2.70). When stratifying by smoking status, presence of varicose veins, type 2 diabetes mellitus and any hormone administration before, no significant differences were found in any groups.

**Conclusion:**

Our study suggested that *APOE* E3/E4 genotype was associated with a higher LEDVT risk. Additional studies are needed to confirm this finding.

## Introduction

The lower extremity deep venous thrombosis (LEDVT) and its complications remain a finding of high incidence in hospitalized patients [1]. The use of color-flow Doppler ultrasound has achieved a higher accuracy in diagnosis of LEDVT than clinical examination alone [1,2]. The advantages of color-flow Doppler ultrasound have also provided conditions to restart investigations concerning the incidence of bilateral LEDVT in patients with a single symptomatic limb [1,3]. LEDVT is a multifactorial medical problem with genetic and acquired risk factors playing in concert in its pathogenesis [4-8]. Strong genetic factors have been implicated in the aetiology of LEDVT [9,10].

Apolipoprotein E (apoE) is a member of apolipoprotein family. *APOE* gene, located on the long arm of chromosome 19, codes for a 299-amino acid protein (apoE). ApoE is a polymorphic glycoprotein involved in cholesterol transport and cell membrane maintenance and repair [11,12]. *APOE* exists in three common allelic forms: E2, E3, and E4, giving six possible genotypes (E2/E2, E2/E3, E2/E4, E3/E3, E3/E4 and E4/E4) [13-15]. *APOE* gene polymorphisms seem to have some impact among patients with cardiovascular disease [16-18].

Recently, a few studies with different results were performed to investigate the association between *APOE* gene polymorphisms and DVT [19,20]. However, its role in the LEDVT has not been well studied. The objective of this study was to investigate the potential association between *APOE* gene polymorphisms and LEDVT.

## Materials and methods

### Study population

A hospital-based case–control study was conducted in 300 patients with LEDVT and 300 age- and gender-matched healthy controls during the years 2010 to 2013 from the institute of ultrasound imaging in the second affiliated hospital of Chongqing Medical University, China. The diagnosis of LEDVT was made by combining the results of color-flow Doppler ultrasound, D-dimer levels, and phlebography. To confirm the diagnosis, two physicians reviewed the hospital records and validated each case. The healthy control subjects were matched with the patients for age and sex. Healthy control subjects were recruited from the Second Affiliated Hospital of Chongqing Medical University where they were attending a clinic for routine examination. The Ethical Committee of the Second Affiliated Hospital of Chongqing Medical University approved the study protocols, and all participants gave written informed consent according to the Declaration of Helsinki.

### DNA extraction and genotyping

The commercially available Qiagen kit (QIAGEN Inc., Valencia, CA, USA) was used to extract DNA from peripheral blood leukocytes. Polymerase chain reaction restriction fragment length polymorphism (PCR-RFLP) assay was applied to assess the *APOE* gene polymorphisms. Based on the GenBank reference sequence, the PCR primers were as follows: sense, ACAGAATTCGCCCCGGCCTGGTACAC; antisense, TAAGCTTGGCACGGCTGTCCAAGGA. The amplified DNA was digested with *Hha* I at 37°C for 2 hours, and analyzed by 6% polyacrylamide gel electrophoresis (PAGE). For quality control, all genotyping personnel were blinded to clinical and imaging data.

### Statistical analysis

The allele and genotype frequencies of *APOE* gene in patients were compared to controls by Chi-square test. The *P* value of statistical significance was adjusted by Fisher’s exact test where appropriate. We applied multivariate logistic regression to calculate crude and adjusted odds ratios (OR) and 95% confidence intervals (CI) for the association between the *APOE* genotypes and LEDVT risk. The Hardy–Weinberg equilibrium was tested for goodness-of-fit chi-square test with one degree of freedom to compare the observed genotype frequencies among the subjects with the expected genotype frequencies. A *P*-value was considered significant at a level of < 0.05. All analyses were performed with Stata software (College Park,Tex.).

## Results

The demographical and clinical features of the participants were showed in Table 1. The mean age was 51.7 (±10.7) years for the LEDVT cases and 52.0 (±11.9) years for the controls. The 63.3% participants were male for the LEDVT cases and 61.7% for the controls. No significant differences were found between the patients and the controls in gender, age, smoking status, presence of varicose veins, type 2 diabetes mellitus and any hormone administration before (Table 1).

**Table 1:** The demographical and clinical features of the participants

	**LEDVT**	**HC**	***P***
Total no.	300	300	
Gender (Male/female)	190/110	185/115	0.67
Age (Year)	51.7 ± 10.7	52.0 ± 11.9	0.75
Smoking status (Ever/never)	122/178	116/184	0.62
Presence of varicose veins (YES/NO)	72/228	55/245	0.09
Type 2 diabetes mellitus (YES/NO)	25/275	14/286	0.07
Any hormone administration before (YES/NO)	30/270	18/282	0.07
Family history of LEDVT (Positive/negative)	58/242	-	-

LEDVT, lower extremity deep venous thrombosis; HC, healthy control.

Patients with LEDVT had a significantly higher frequency of *APOE* E3/E4 genotype (OR =1.48, 95% CI = 1.05, 2.10; *P* = 0.03) than healthy controls (Table 2). When stratifying by family history of LEDVT, it was found that patients with positive family history of LEDVT had a significantly higher frequency of *APOE* E3/E4 genotype (OR =1.68, 95% CI = 1.04, 0.95; *P* = 2.70) (Table 3). When stratifying by smoking status, presence of varicose veins, type 2 diabetes mellitus and any hormone administration before, no significant differences were found in any groups (Table 3).

**Table 2:** **Frequencies of *APOE *** **gene polymorphisms in LEDVT and HC groups**

**Genotype**	**LEDVT n (%)**	**HC n (%)**	**OR (95% CI)**	***P***
E2/E2	3(1.0)	2(0.7)	1.51(0.25,9.07)	0.66
E2/E3	1(0.3)	2(0.7)	0.50(0.05,5.53)	0.57
E2/E4	1(0.3)	2(0.7)	0.50(0.05,5.53)	0.57
E3/E3	185(61.7)	206(68.7)	0.73(0.52,1.03)	0.07
E3/E4	105(35.0)	80(26.6)	1.48(1.05,2.10)	0.03
E4/E4	5(1.7)	8(2.6)	0.62(0.20,1.91)	0.40
E2 allele frequency	8(1.3)	8(1.3)	1.00(0.37,2.68)	1.00
E3 allele frequency	476(79.3)	494(82.3)	0.82(0.62,1.10)	0.19
E4 allele frequency	116(19.3)	98(16.3)	1.23(0.91,1.65)	0.18

LEDVT, lower extremity deep venous thrombosis; HC, healthy control; OR, odds ratio; CI, confidence interval.

**Table 3:** **Stratification analysis of *APOE *** **genotype frequency in LEDVT patients**

**Variable**	**Cases**	**E2/E2**		**E2/E3**		**E2/E4**		**E3/E3**		**E3/E4**		**E4/E4**	
		**n (%)**	***P***	**n (%)**	***P***	**n (%)**	***P***	**n (%)**	***P***	**n (%)**	***P***	**n (%)**	***P***
Smoking status	300	3(1.0)		1(0.3)		1(0.3)		185(61.7)		105(35.0)		5(1.7)	
Ever	122	1(0.8)	0.86	0(0)	0.90	0(0)	0.90	71(58.2)	0.74	48(39.3)	0.57	2(1.6)	0.98
Never	178	2(1.1)	0.90	1(0.6)	0.71	1(0.6)	0.71	114(64.0)	0.80	57(32.0)	0.64	3(1.7)	0.99
Presence of varicose veins	300	3(1.0)		1(0.3)		1(0.3)		185(61.7)		105(35.0)		5(1.7)	
YES	72	1(1.4)	0.78	0(0)	0.84	0(0)	0.84	45(62.5)	0.95	25(34.7)	0.98	1(1.4)	0.87
NO	228	2(0.9)	0.89	1(0.4)	0.85	1(0.4)	0.85	140(61.4)	0.98	80(35.1)	0.99	4(1.8)	0.94
Type 2 diabetes mellitus	300	3(1.0)		1(0.3)		1(0.3)		185(61.7)		105(35.0)		5(1.7)	
YES	25	1(4.0)	0.24	0(0)	0.41	0(0)	0.41	16(64.0)	0.91	7(28.0)	0.61	1(4.0)	0.43
NO	275	2(0.7)	0.73	1(0.4)	0.95	1(0.4)	0.95	169(61.5)	0.98	98(35.6)	0.91	4(1.4)	0.84
Any hormone administration before	300	3(1.0)		1(0.3)		1(0.3)		185(61.7)		105(35.0)		5(1.7)	
YES	30	1(3.3)	0.30	0(0)	0.47	0(0)	0.47	18(60.0)	0.93	9(30.0)	0.70	2(6.7)	0.11
NO	270	2(0.7)	0.74	1(0.4)	0.94	1(0.4)	0.94	167(61.9)	0.98	96(35.5)	0.92	3(1.1)	0.58
Family history of DVT	300	3(1.0)		1(0.3)		1(0.3)		185(61.7)		105(35.0)		5(1.7)	
Positive	58	1(1.7)	0.64	0(0)	0.74	0(0)	0.74	22(37.9)	0.07	34(58.6)	0.04	1(1.7)	0.98
Negative	242	2(0.8)	0.84	1(0.4)	0.88	1(0.4)	0.88	163(67.4)	0.52	71(29.3)	0.32	4(1.7)	0.99

## Discussion

Many studies have been performed to investigate an association of genetic polymorphism and DVT. A case–control study found that subjects carrying endothelial protein C receptor (EPCR) gene 6936AG genotype likely had an increased risk of DVT [21]. The 20210 A allele of the prothrombin gene was a common risk factor among Swedish outpatients with verified DVT [22]. A case control study found an excess of rare coding single-nucleotide variants of the ADAMTS13 gene in patients with DVT [23]. A homozygosity state for 20210A prothrombin variant in a young woman was a cause of DVT during pregnancy [24]. Susceptibility to DVT in North Indian Asian patients may be associated with some variants of nitric oxide synthase 3 (NOS3) gene [25]. A large case–control study found that factor XIII (FXIII) 34Leu carriers were associated with a slightly decreased DVT risk [26]. A case control study found that genetic variants in the F11 gene were risk factors for DVT among both Whites and Blacks [27]. A meta-analysis indicated that rs2227589 in the SERPINC1 gene, rs13146272 in the CYP4V2 gene and rs1613662 in the GP6 gene were risk factors for DVT among Whites [27]. A case control study concluded that the G534E-polymorphism of the gene encoding the factor VII-activating protease is a risk factor for DVT [28]. A case control study suggested that tumor necrosis factor alpha (TNF-alpha) -308A allele was association with the risk of DVT [29]. The rs4524 SNP in F5 was consistently associated with DVT in 3 large case–control studies [30]. A case control study suggested that factor V G1691A (Leiden) and A4070G (HR2 Haplotype) polymorphisms were independent risk factors for DVT [31,32].

The *APOE* gene polymorphisms were associated with many other diseases. The E4 allele of the *APOE* gene was associated with a worse lipid profile in the Brazilian urban population [33]. In the Tunisian population the *APOE* E4 appears to be only indirectly involved in the severity of coronary artery disease [34]. E2 allele of the *APOE* gene polymorphism was predictive for obesity status in Roma minority population of Croatia [35]. A meta-analysis included 29 studies involving 2,737 CI cases and 2,689 controls suggested that *APOE* E4 allele was associated with an increased risk of developing cerebral infarction in Chinese population [36]. A meta-analysis of 45 studies including 13,940 cases and 16,364 controls found that *APOE* gene polymorphisms were associated with essential hypertension [37]. A meta-analysis of 28 case–control studies provided evidence for an association between the *APOE* E4 allele and frontotemporal lobar degeneration [38]. A meta-analysis of seven studies, including 2,090 cases and 742 control suggested an association between *APOE* E4 mutation and increased risk of recurrent pregnancy loss [39]. A meta-analysis of seven studies with 966 patients and 1,086 controls suggested that the *APOE* polymorphisms were assoated with the risk of psoriasis, especially E2 and E3 alleles [40]. A meta-analysis of experimental and human studies suggested an association between *APOE* gene expression and its gene polymorphism with nephrotic syndrome susceptibility [41]. A meta-analysis of 6977 subjects provides evidence that *APOE* E2 mutation is associated with multiple sclerosis risk [42]. A meta-analysis of 29 studies included 1,763 cases and 4,534 controls provided evidence for an association between *APOE* gene polymorphisms and the risk of vascular dementia [43]. A meta-analysis of 17 studies, including 1,773 cases and 2,751 controls, revealed that *APOE* gene E4 allele was a risk factor of gallbladder stone disease, especially in elder people and Chinese population [44].

Some limitations of this study should be noted. First of all, this is a hospital based case control study, so the selection bias cannot be avoidable and the subjects may not be representative of the general population. Second, the sample size of this study is relatively small, which may not have enough statistical power to explore the real association. Third, these results should be interpreted with caution because the population was only from China, which reduces the possibility of confounding from ethnicity, so it does not permit extrapolation of the results to other ethnic groups.

## Conclusion

In conclusion, our study suggested that *APOE* E3/E4 genotype was associated with a higher LEDVT risk. When stratifying by family history of LEDVT, it was found that patients with positive family history of LEDVT had a significantly higher frequency of *APOE* E3/E4 genotype. Additional studies are needed to confirm this finding.

## Competing interest

The authors declare that they have no competing interests.

## Authors’ contributions

SLZ and ZGW carried out the molecular genetic studies and drafted the manuscript. XPW and YS carried out the genotyping. YS, DXL participated in the design of the study and performed the statistical analysis. SLZ, ZGW conceived of the study, and participated in its design and coordination and helped to draft the manuscript. All authors read and approved the final manuscript.
